# Electric field induced structural colour tuning of a silver/titanium dioxide nanoparticle one-dimensional photonic crystal

**DOI:** 10.3762/bjnano.7.131

**Published:** 2016-10-06

**Authors:** Eduardo Aluicio-Sarduy, Simone Callegari, Diana Gisell Figueroa del Valle, Andrea Desii, Ilka Kriegel, Francesco Scotognella

**Affiliations:** 1 Center for Nano Science and Technology@PoliMi, Istituto Italiano di Tecnologia, Via Giovanni Pascoli 70/3, 20133 Milan, Italy; 2 Dipartimento di Chimica, Materiali e Ingegneria Chimica "Giulio Natta", Politecnico di Milano, Piazza Leonardo da Vinci 32, 20133 Milano, Italy; 3 Dipartimento di Fisica, Politecnico di Milano, Piazza Leonardo da Vinci 32, 20133 Milano, Italy; 4 Department of Nanochemistry, Istituto Italiano di Tecnologia (IIT), via Morego 30, 16163 Genova, Italy,; 5 Istituto di Fotonica e Nanotecnologie CNR, Piazza Leonardo da Vinci 32, 20133 Milano

**Keywords:** electro-optic switching, photonic crystal, plasmonic nanoparticles

## Abstract

An electric field is employed for the active tuning of the structural colour in photonic crystals, which acts as an effective external stimulus with an impact on light transmission manipulation. In this work, we demonstrate structural colour in a photonic crystal device comprised of alternating layers of silver nanoparticles and titanium dioxide nanoparticles, exhibiting spectral shifts of around 10 nm for an applied voltage of only 10 V. The accumulation of charge at the metal/dielectric interface with an applied electric field leads to an effective increase of the charges contributing to the plasma frequency in silver. This initiates a blue shift of the silver plasmon band with a simultaneous blue shift of the photonic band gap as a result of the change in the silver dielectric function (i.e. decrease of the effective refractive index). These results are the first demonstration of active colour tuning in silver/titanium dioxide nanoparticle-based photonic crystals and open the route to metal/dielectric-based photonic crystals as electro-optic switches.

## Introduction

Structural colour is colour due to the Bragg reflection (in photonic structures for example) as opposed to colour from pigments or colour centres [1]. The active tuning of the structural colour in photonic crystals is a subject that has attracted a great attention in the last decades. The electric field is probably the simplest external stimulus that can be employed for such colour tuning. A recent review article has reported the most important achievements in the electrically driven tunability of photonic crystals [2]. In this interesting article, different types of tuning techniques are encompassed, for example: i) smart polymers [3–7], ii) liquid crystals [8–12], and electrophoresis [13–16].

The employment of metallic nanoparticles for the structural colour tuning with electric field, to the best of our knowledge, has not been reported in the literature. However, plasmon peak tuning of gold nanoparticles with an electric field in an electrochemical cell has been recently shown [17], opening the way to a new strategy for electro-optical switches with metal nanostructures.

In this paper we show experimental evidence of structural colour tuning with an electric field in a one-dimensional photonic crystal made of alternating layers of silver nanoparticles and titanium dioxide nanoparticles. We have observed a blue shift of about 10 nm with an applied voltage of 10 V. We give an interpretation of the phenomenon based on the increased carrier density participating in the plasma frequency of silver. Such charges are due to the polarization at the titanium dioxide/silver interface upon application of an electric field.

## Results and Discussion

**Figure 1:**
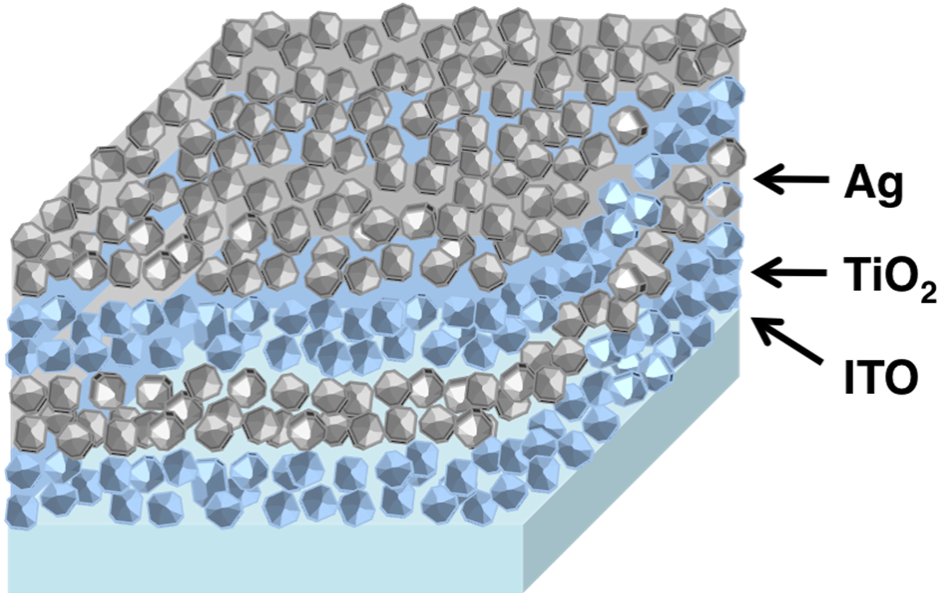
Scheme of the one-dimensional photonic crystal made of layers of silver nanoparticles and titanium dioxide nanoparticles. The actual photonic crystal fabricated in this work is composed of five silver/titanium dioxide bilayers.

The fabricated photonic crystal is made of five bilayers of silver nanoparticles and titanium dioxide nanoparticles deposited on top of an indium tin oxide (ITO) substrate. A scheme of the photonic crystal is shown in Figure 1.

The silver nanoparticles have a diameter of about 50 nm, while the TiO_2_ nanoparticles are smaller than 15 nm. The surface topography and phase atomic force microscopy (AFM) images of a Ag layer, a TiO_2_ layer and the top surface of a five bilayer Ag/TiO_2_ photonic crystal, all deposited on glass/ITO substrates, are presented in the Supporting Information File 1 (Figure S1) and show that the silver nanoparticle layer has the highest surface roughness among the different samples. This is due to the large size of the silver nanoparticles and the formation of large aggregates. Notably, the lowest surface roughness was found for the top TiO_2_ surface layer of the five bilayer photonic crystal (Figure S1c of Supporting Information File 1). The formation of a more compact layer with a reduced surface roughness is most probably a result of the small TiO_2_ nanoparticles deposited onto the Ag films, which fill the empty voids between the Ag aggregates and promote a certain degree of intermixing between the two different types of nanoparticles at the Ag/TiO_2_ interfaces. Indeed, scanning electron microscopy (SEM) images (Figure S2 in Supporting Information File 1) of the cross section of a photonic crystal shows five bilayers (the single silver and TiO_2_ layers cannot be distinguished due to the resolution of our instrument) of a total thickness of 600 nm. This value is lower than the sum of five single Ag and TiO_2_ layers deposited directly on the substrate (60 nm and 120 nm, respectively), confirming the conclusions of the AFM analysis. Nevertheless, we consider that this intermixing is confined to the interface region and that a bilayer structure is still obtained with a blurred interface.

**Figure 2:**
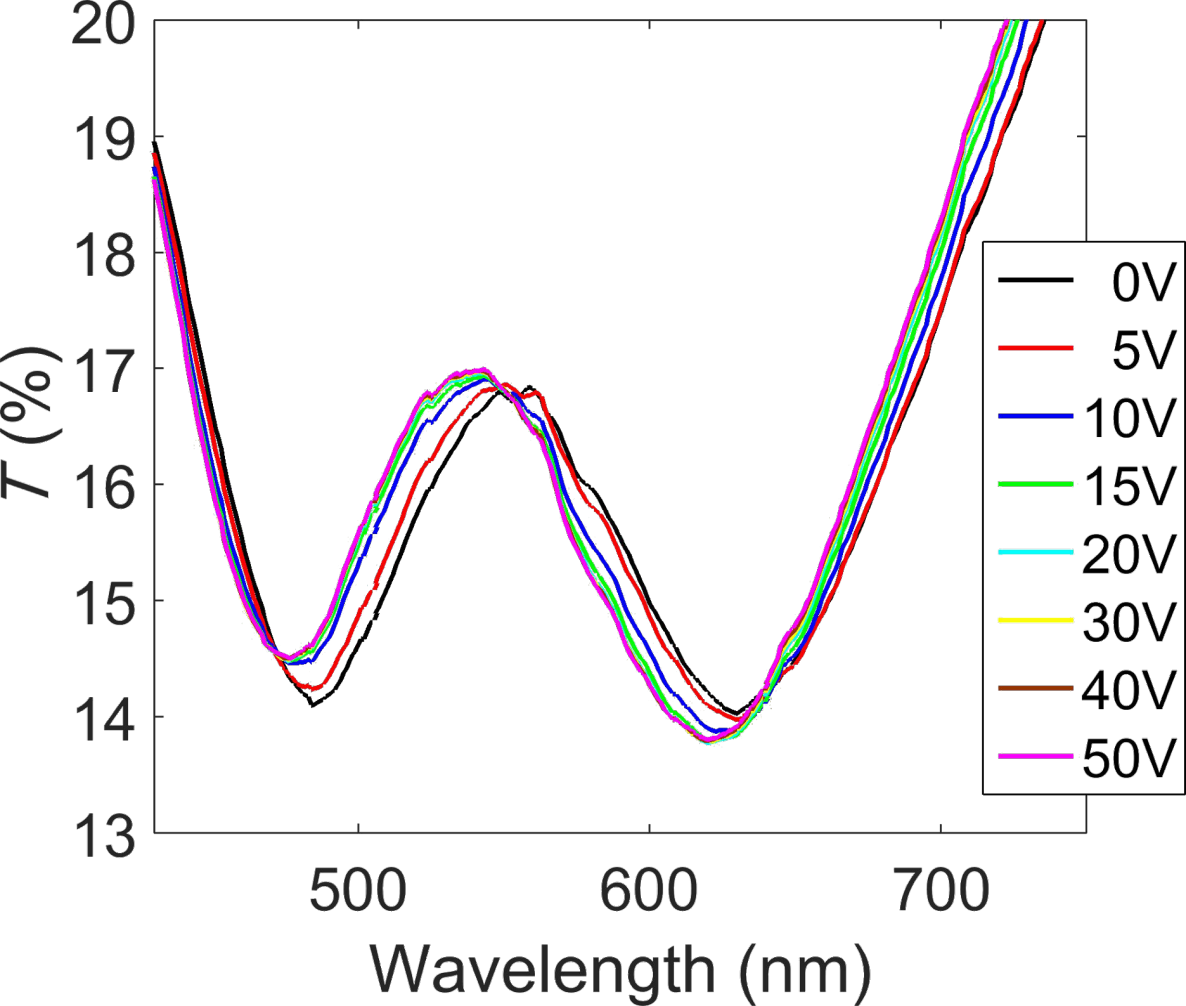
Transmission spectra of the ITO–(Ag nanoparticle/TiO_2_ nanoparticle)_5_–ITO photonic crystal device upon application of an electric field.

For the electro-optical characterization, we placed another ITO substrate on the other side of the photonic crystal and applied an external voltage to provide an electric field to the photonic crystal device. The electro-optical measurement is shown in Figure 2, where the transmission spectrum of the photonic crystal is reported as a function of the applied voltage. The transmission is dominated by two strong bands at around 480 nm and 620 nm, ascribed to the plasmonic resonances of the silver nanoparticles and the photonic bandgap, respectively.

**Figure 3:**
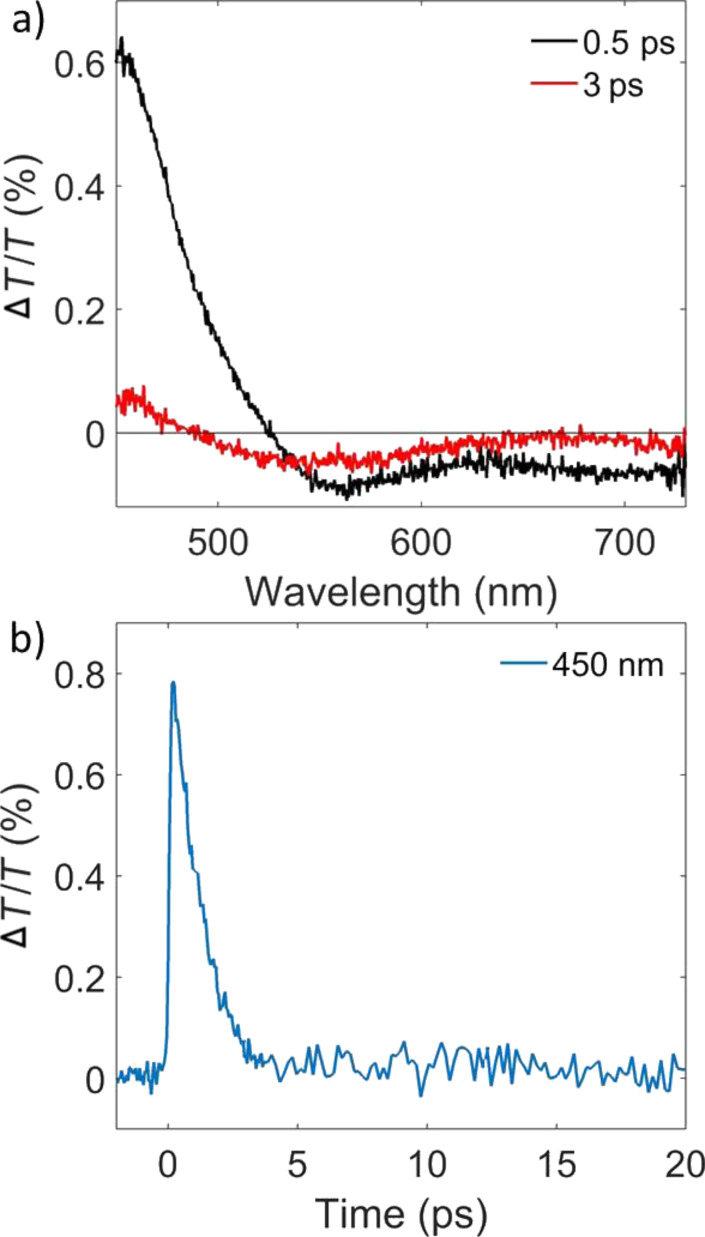
(a) Pump–probe spectra at different time delays and (b) pump–probe dynamic at 450 nm of the ITO–(Ag nanoparticle/TiO_2_ nanoparticle)_5_–ITO photonic crystal.

We want to emphasize the fundamentally different nature of the two resonances observed in our device, namely the plasmonic resonance of the silver nanoparticle layer and that of the photonic bandgap. The pump–probe measurement in Figure 3a shows the transmission spectra of the transient absorption measurements at delay times of 500 and 3000 fs (black and red curve, respectively). We observe the typical plasmonic response of the silver nanoparticles as a derivative shape of the peak at 480 nm (Figure 3a). The temporal behaviour of metallic nanoparticles is characterized by three different regimes [18]: i) the pump excitation strongly perturbs the Fermi distribution and creates electrons that are not in thermal equilibrium, called energetic electrons; via electron–electron scattering, within a few tens to hundreds of fs, a new Fermi distribution of hot electrons is obtained. ii) Within a few ps, the hot electrons release their energy to the lattice via electron–phonon scattering. iii) The hot lattice releases its energy to the environment within hundreds of ps. With the temporal resolution of our setup, which is about 150 fs, we could not observe the electron–electron scattering, but the dynamic of the resonance at 450 nm reported in Figure 3b is related to the electron–phonon scattering. This picosecond-scale dynamic is followed by a very weak phonon–phonon scattering. The photonic band gap (around 620 nm) does not show any particular dynamic (not shown here), as expected. The combination of a metal and a dielectric in the photonic device is a key to the voltage-dependent observations, as will be explained later in this manuscript.

**Figure 4:**
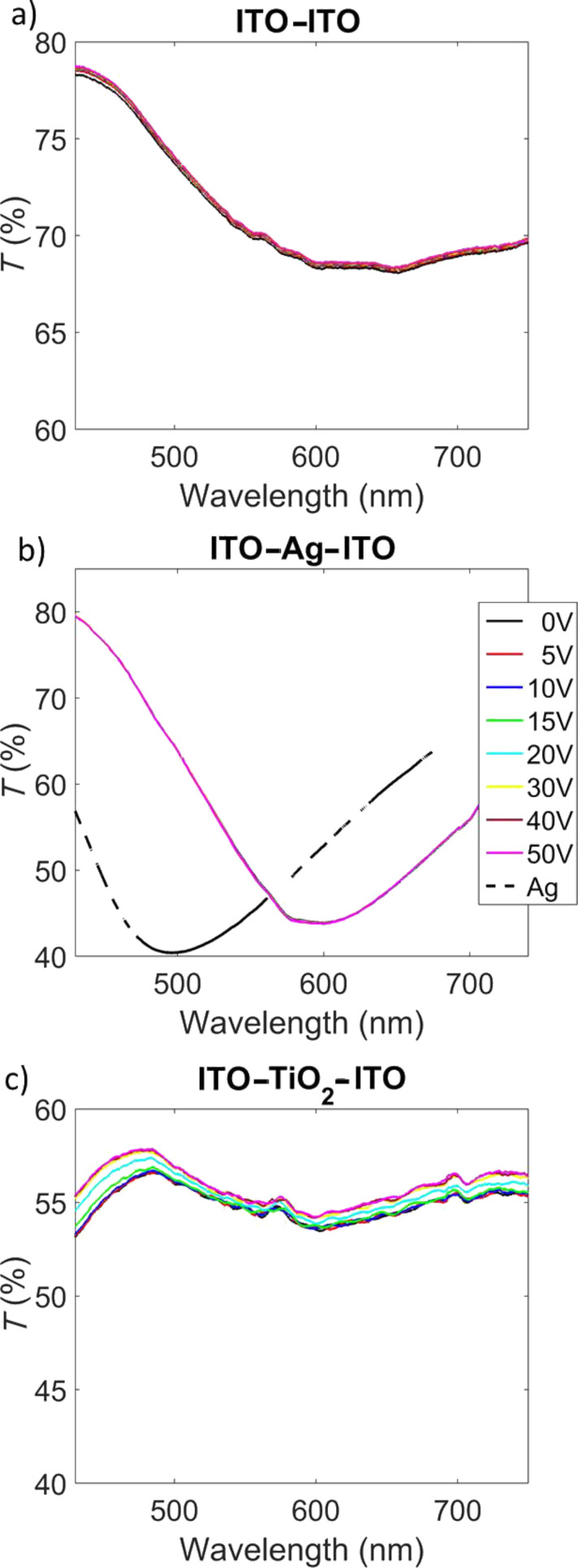
(a) ITO–ITO device under electric field; (b) ITO–Ag nanoparticle–ITO device under electric field; (c) ITO–TiO_2_ nanoparticle–ITO device under electric field.

Upon application of an electrical potential to the device, we observe a blue shift of the entire transmission spectrum, that is, of the photonic band gap as well as the plasmon resonance of the silver nanoparticles. The shift of the photonic band gap is about 10 nm for an applied potential of only 10 V. In Figure S7 of Supporting Information File 1 we show that at voltages above 15 V the shift saturates up to a value of about 16–17 nm. Notably, the observed shifts of both resonances are a result of the alternation between the metal and the dielectric nanoparticle layer, as affirmed by several counter experiments, as demonstrated in Figure 4.

We investigated three scenarios: first, the results for devices with only ITO substrates placed together, as given in Figure 4a; second, the results of only the silver layer between two ITO substrates, as given in Figure 4b; and third, for only the titanium dioxide layer between two ITO substrates (Figure 4c). For all three investigated cases, the observed spectral changes by applying a potential to the device are negligible, even in the region of the silver plasmon band (Figure 4b). The strong red shift of the plasmonic peak when the silver nanoparticle layer is deposited on ITO (620 nm) with respect to the glass substrate (480 nm) was observed in the static samples (i.e. without applying the voltage is ascribed to a coupling between the high carrier density of ITO and the silver nanoparticle plasmon). A difference in fill factor might also play a role here, leading to a stronger coupling and an intense red shift. Nevertheless, these results demonstrate that the observed shifts with applied voltage are only observed in the alternating silver and titanium dioxide nanoparticle layers.

**Figure 5:**
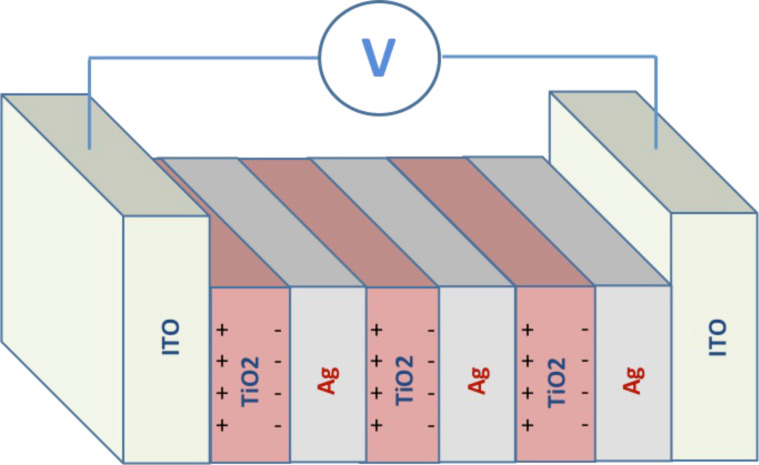
Scheme of the interpretation of the action of the electric field on the ITO–(Ag nanoparticle/TiO_2_ nanoparticle)_5_–Ag–ITO photonic crystal device.

In the following we provide an interpretation for the observed blue shift of the photonic band gap as well as the silver plasmon resonance by applying an electric field and making a simple assumption. We consider the plasma frequency ω_p_ for silver as

[1]
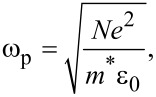


where *N* is the carrier density, *e* is the electron charge, m* is the effective mass and ε_0_ is the dielectric constant of the vacuum. Qualitatively, we can state that the polarization charges that accumulate at the silver/titanium dioxide interface, because of the electric field, effectively increase the carrier density involved in the plasma frequency, as schematically depicted in Figure 5. Moreover, a second contribution can be the flow of charges in the silver layers due to the electric field itself. In this way, we have the carrier density with the electric field (*N*^E^) versus the initial carrier density (*N*), such that *N*^E^ > *N*.

The Drude model can be used to predict the behaviour of the plasmonic response in the photonic crystal [19]. The frequency-dependent complex dielectric function of silver can be written as

[2]
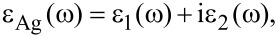


where

[3]
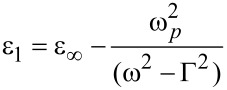


and

[4]
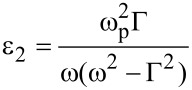


with Γ representing the free carrier damping [20].

The dielectric function of the silver nanoparticle film (a network of necked silver nanoparticles with air pores) can be described by the Maxwell–Garnett effective medium approximation [21–23], which is given by

**Figure 6:**
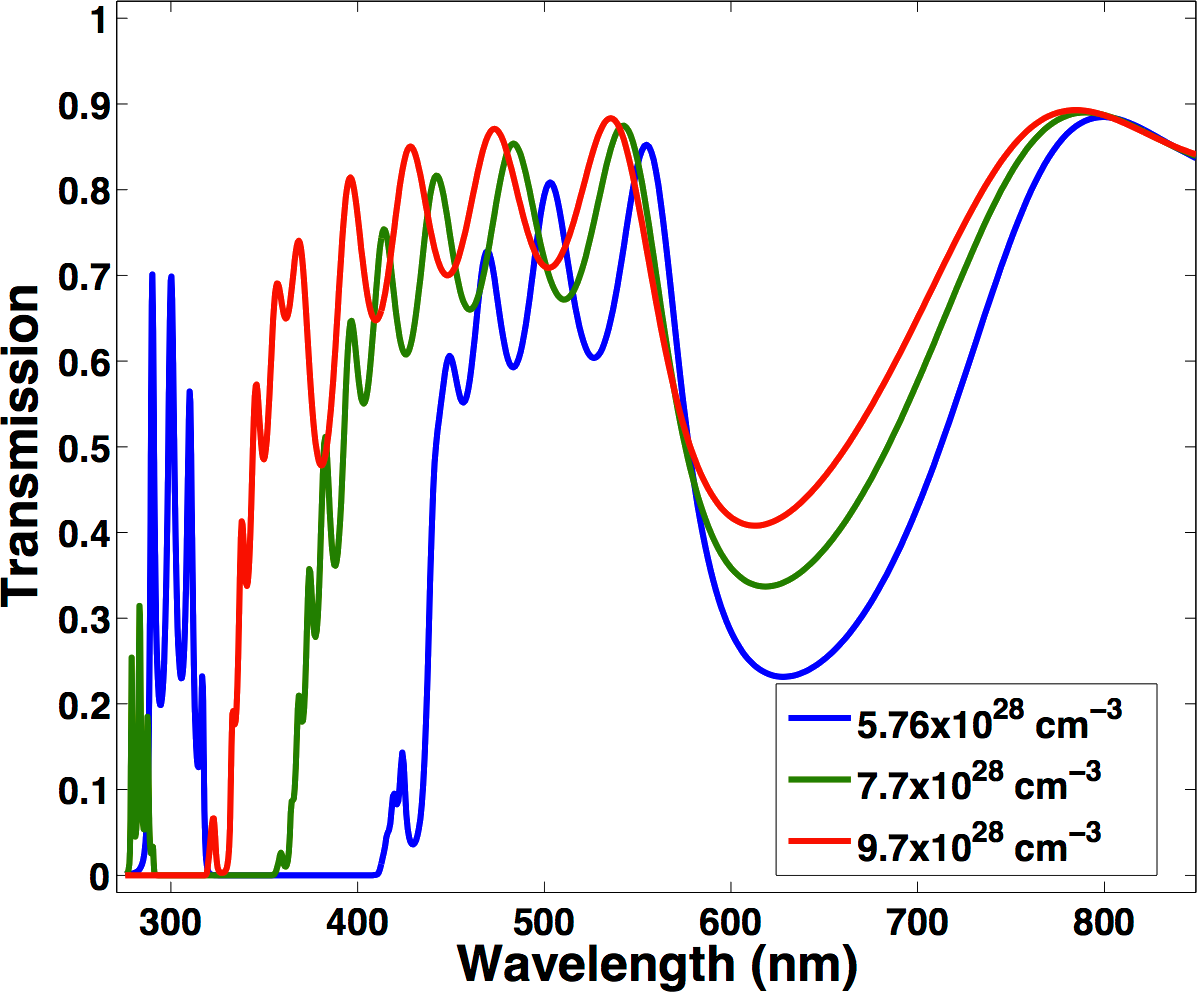
Transmission spectra simulated with the transfer matrix method for a Ag/TiO_2_ nanoparticle photonic crystal device with three different carrier densities.

[5]
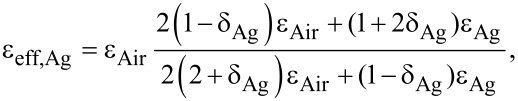


where ε_Air_ is the dielectric constant of air, and δ_Ag_ accounts for the volume fraction occupied by the silver nanoparticles. Given *n*_eff,Ag_^2^ = ε_eff,Ag_ and taking the refractive index dispersion of titanium dioxide from the literature [24–25] we used the transfer matrix method [26–27] to simulate the transmission spectrum of the photonic crystal. We remark here that the dielectric function of both the Ag and the TiO_2_ nanoparticle layers were calculated with the effective medium approximation, as is thoroughly discussed in Supporting Information File 1. In Figure 6 the results of the calculation for three different carrier densities are given, where blue is the actual carrier density of Ag as given in [28] and two artificially increased carrier densities. Similar to the experimental results, the calculated transmission spectra show an intense band in the UV/blue region ascribed to the plasmon resonance of the silver layer and a second band corresponding to the photonic bandgap. Note that the plasmon resonance of silver overlaps with some thin film interference features (see Figure S4 in the Supporting Information File 1). To unambiguously assign the plasmon resonance, we performed simulations to distinguish the absorption contribution (i.e. the imaginary part of the refractive index of Ag, see Figure S5 in the Supporting Information File 1). From the simulation of the spectra, we see that an increase of the carrier density induces a blue shift of the photonic band gap, confirming the interpretation of our experimental findings.

We used the same model to estimate the number of charges added to the system. Without the applied voltage (Supporting Information File 1, Figure S3), we used the parameters for the silver dielectric function as reported in [28] with the electron density of silver *N* = 5.76 × 10^28^ m^−3^ to simulate the optical properties of the multilayer structure. To get an estimate of the number of charges introduced with an applied voltage of 40 V (Supporting Information File 1, Figure S6), we had to increase the carrier density to the value of *N*^E^ = 6.86 × 10^28^ m^−3^. This resulted in a difference of Δ*N* = 1.1 × 10^28^ m^−3^. Taking into account that the five silver layers have a volume of (60 × 10^−9^ m × 5) × 0.015 m × 0.015 m, and that the diameter of the Ag nanoparticles is about 50 nm, we could estimate an increase of about 10^5^ charges per particle. We remark here that this discussion just gives a rough estimate of the required number of charges added to each nanoparticle to induce the shifts observed. Indeed, in the model, we assumed for simplicity a change in the carrier density over the entire volume of the nanocrystal, although, as studied in [17] , charge accumulation in metallic nanoparticles occurs for diameters around 5 nm only (in contrast to the 50 nm diameter). An exact evaluation would require a deeper analysis. Moreover, Brown et al. [17] showed that the electrochemical doping of gold nanoparticles in solution is, apart from a change in carrier density, accompanied by an increase in the surrounding medium refractive index. A change of the dielectric surrounding, however, also largely influences the position of the plasmon resonance [29]. In addition, doping occurs only in a thin layer at the nanoparticle surface. These two effects also ultimately impact on the carrier damping, which in our estimation was kept constant. Studies on the accumulation of charges in an ITO film by applying a constant voltage demonstrated that besides an increase in carrier density, other Drude parameters such as the damping constant and the high frequency dielectric constant are altered through the introduction of additional carriers [30]. Thus, a deeper study of the effect of applied voltage on thin films of Ag would be required for an exact evaluation of the effect on the Drude parameters and a more precise extraction of the number of carriers injected. Nevertheless, our estimation is in good agreement with results on the electrochemical doping of Au nanoparticles in solution, as observed by Ung et al [31]. Here the injection of 1600 ± 300 carriers was found in nanoparticles of 11.5 nm in diameter, which corresponds to an increase in the carrier density by around Δ*N* = 2 × 10^27^ m^−3^. This value is about one order of magnitude below our findings, corresponding to the order of magnitude lower carrier density of Au with respect to Ag.

## Conclusion

In this work we studied the tuning of the structural colour, that is, the active shift of the photonic band gap, in a one-dimensional photonic crystal made of alternating silver nanoparticle and titanium dioxide nanoparticle layers. A concomitant blue shift of the silver plasmon peak and of the photonic band gap of about 10 nm with a 10 V applied voltage was observed. We have proposed an interpretation of this observation in this article: the electric field induces the accumulation of polarization charges at the silver/titanium dioxide interface. These charges contribute to the plasma frequency of silver, which due to the porosity of the layer and the subsequent high surface/volume ratio, will allow the electron density to increase over the entire volume of the silver layer, resulting in an increase of the carrier density and a blue shift of the plasma frequency. We estimated an increase in carrier density by Δ*N* = 1.1 × 10^28^ m^−3^. Consequently, the effective refractive index of the whole photonic crystal is also changed, leading to the blue shift of the photonic band gap. Our results highlight the possibility to employ these photonic structures to manipulate the transmission of light.

## Methods

### Nanoparticle colloidal dispersions

Silver nanoparticle dispersion was purchased by Sigma-Aldrich and was diluted in triethylene glycol monoethyl ether (Sigma-Aldrich) up a final concentration of 5 wt %. The diameter of the nanoparticles was less than 50 nm. The TiO_2_ sol was synthesized by following a protocol reported in the literature based on the hydrolysis of titanium tetraisopropoxide (Ti(OCH_2_CH_2_CH_3_)_4_ (TTIP, 97%, purchased from Sigma-Aldrich) [32]. Briefly, a mixture of 2.5 mL of ethanol and 15 mL of TTIP was added dropwise, in a three-neck round bottom flask, to 90 mL of distilled water to obtain a TTIP/ethanol/water mixture with a molar ratio of 1:0.75:83. Subsequently, 1 mL of hydrochloric acid (purchased by Sigma-Aldrich) was added and the obtained sol was refluxed under stirring for 8 h at 80 °C, resulting in a stable, milky solution. Before layer deposition, we concentrated the nanoparticle dispersion in order to make thicker layers.

### Photonic crystal fabrication

The photonic crystal was fabricated on an indium tin oxide (ITO) substrate using a spin coater (Laurell, WS-400- 6NPP-Lite). The rotation speeds for the deposition were 2000 rpm and 2000 rpm for silver and titanium dioxide nanoparticles, respectively. After each deposition, the sample was annealed for 10 min at 350 °C on a hot plate under the fume hood.

### Optical measurements with electric field

The photonic crystal, fabricated on ITO substrate, was covered with another ITO substrate in order to apply an electric field. To apply an electric field, we employed a simple voltage supply with a 100× amplifier. The transmission spectra were collected with a Shimazdu spectrophotometer.

### Pump–probe experiment

For this experiment, an amplified Ti:sapphire laser system was employed (150 fs pulse duration, 1 kHz repetition rate, 800 nm excitation wavelength). The pump pulse at 400 nm was achieved via second harmonic generation. The light transmission was probed with broadband supercontinuum generation in sapphire. The signal was collected by a fast CCD camera connected to a spectrometer and was presented as the differential transmission ΔT/T [33].

## Supporting Information

File 1 Morphological characterization of the sample with atomic force microscopy and scanning electron microscopy; short description of the transfer matrix method; additional simulations of the light transmission in bare silver films and in the photonic crystal.
