# Supplementary material for: Electric field induced structural colour tuning of a silver/titanium dioxide nanoparticle one-dimensional photonic crystal
Source: Beilstein J Nanotechnol. 2016 Oct 6;7:1404–10. doi: 10.3762/bjnano.7.131 (PMC5082530; doi:10.3762/bjnano.7.131)
Supplement: File 1 — Morphological characterization of the sample with atomic force microscopy and scanning electron microscopy; short description of the transfer matrix method; additional simulations of the light transmission in bare silver films and in the photonic crystal. [file Beilstein_J_Nanotechnol-07-1404-s001.pdf]

Supporting Information

for

Electric field induced structural colour tuning of a silver/titanium dioxide nanoparticle one-dimensional photonic crystal

Eduardo Aluicio-Sarduy[1,2], Simone Callegari[2], Diana Gisell Figueroa del Valle[1,3], Andrea Desii[1], Ilka Kriegel[4], and Francesco Scotognella[1,3,5,*]

Address: [1]Center for Nano Science and Technology@PoliMi, Istituto Italiano di Tecnologia, Via Giovanni Pascoli 70/3, 20133 Milan, Italy, [2]Dipartimento di Chimica, Materiali e Ingegneria Chimica "Giulio Natta", Politecnico di Milano, Piazza Leonardo da Vinci 32, 20133 Milano, Italy, [3]Dipartimento di Fisica, Politecnico di Milano, Piazza Leonardo da Vinci 32, 20133 Milano, Italy, [4]Department of Nanochemistry, Istituto Italiano di Tecnologia (IIT), via Morego 30, 16163 Genova, Italy, and [5]Istituto di Fotonica e Nanotecnologie CNR, Piazza Leonardo da Vinci 32, 20133 Milano

Email: Francesco Scotognella - francesco.scotognella@polimi.it

* Corresponding author

# Morphological characterization and additional light transmission simulations.

Introduction

In this Supporting Information we report the characterization of the photonic crystal by atomic force microscopy (AFM) and scanning electron microscopy (SEM). Then we describe in detail the transfer matrix method used to simulate the transmission spectrum of the photonic crystal and provide a discussion on the results.

Morphological characterization

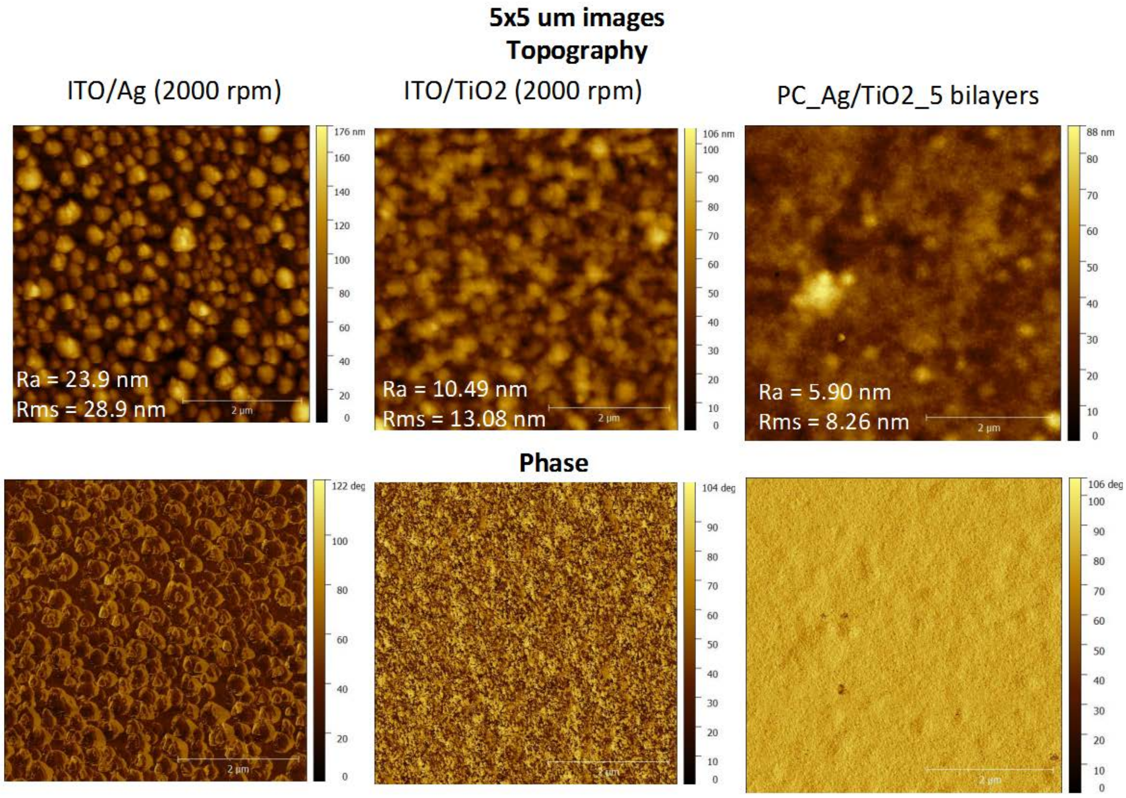

Figure S1: Atomic force microscopy height and phase images of (a) silver nanoparticles film, (b) TiO_2 nanoparticles film and (c) top surface of a five bilayers Ag/TiO_2 photonic crystal. All the films were deposited on glass/ITO substrates.

Figure S1 shows the surface topography and phase AFM images of an Ag layer, a TiO_2 layer and the top surface of a 5 bilayer Ag/TiO_2 photonic crystal, deposited on glass/ITO substrates. The AFM images have been obtained using an Agilent 5500 in tapping mode under ambient conditions. As it can be noticed, the roughness of the silver nanoparticles film is the highest among the different samples measured. The large size of the silver nanoparticles (about 50 nm) results in the formation of large aggregates with the consequent impact on the surface roughness as it can be seen in Figure S1a. Instead, for the TiO_2 layer (Figure S1b) the lower size of the nanoparticles (lower than 15 nm) allows us to obtain of a more homogeneous layer with a Ra value around 10 nm, less than half of the one for the silver film (23.9 nm). Surprisingly, an even lower Ra value of 5.90 nm is observed on the top TiO_2 surface of the five bilayer photonic crystal (Figure S1c). We consider that the small size TiO_2 nanoparticles deposited onto the Ag films fill the empty spaces between the different Ag aggregates, promoting the formation of a more compact layer with a reduced surface roughness. As a result can be expected a certain degree of intermixing between the two different types of nanoparticles at the Ag/TiO_2 interfaces. Nevertheless, we consider that this intermixing is confined to the interface region and that a bilayer structure is still obtained with a blurred interface.

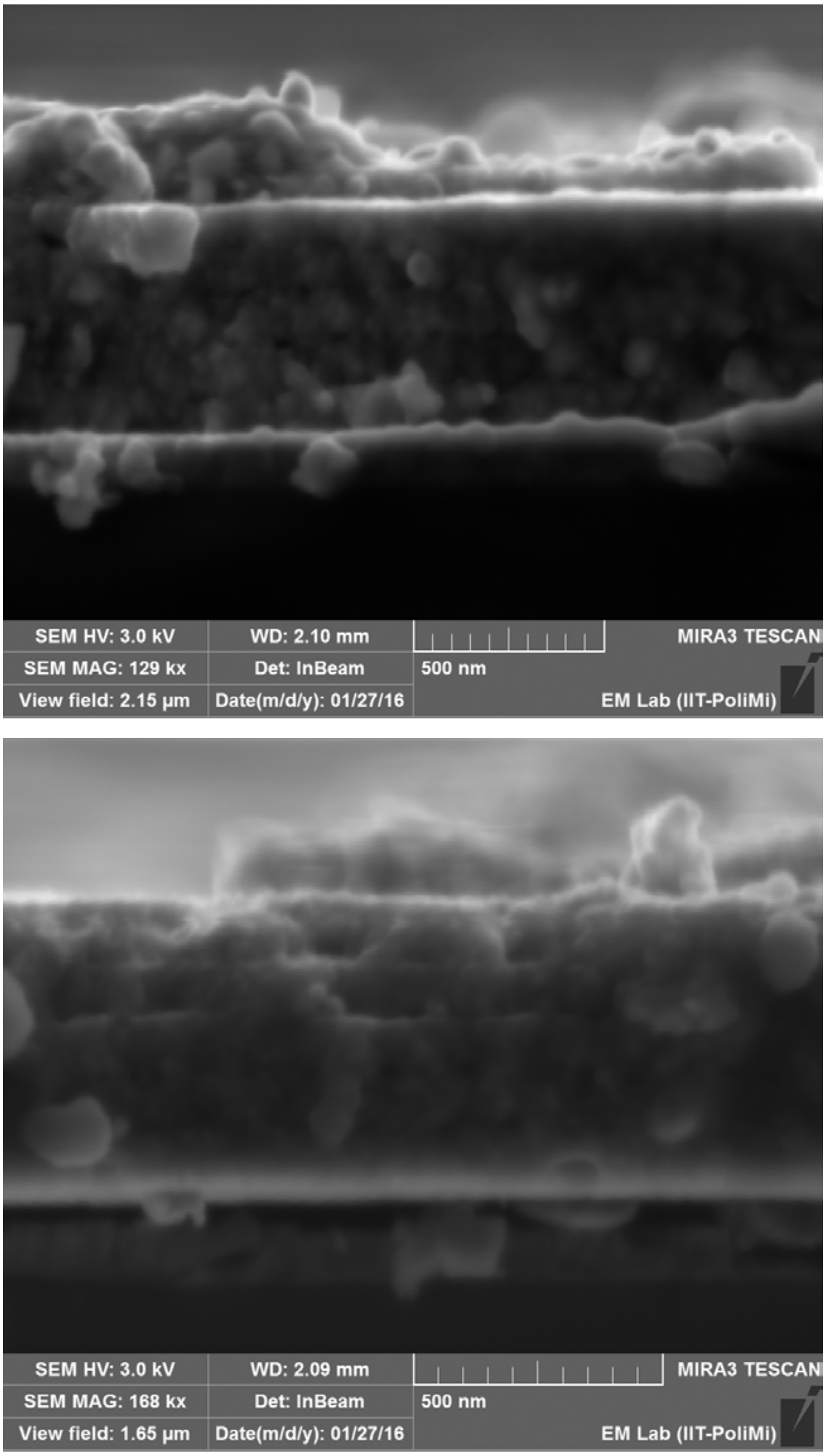

Figure S2: Two SEM cross section images of the (Ag nanoparticle/TiO_2 nanoparticle)$_5$ photonic crystal.

In Figure S2 we show the SEM cross section images of the photonic crystal. The SEM equipment that we have used did not allow us to achieve high resolution images, i.e. enough contrast to observe the independent layers within the bilayers (also due to the intermixing of the components). However, it is possible to identify the 5 Ag nanoparticle/TiO_2 nanoparticle

bilayers of the stack corroborating the presence of ordered structures. For these cross section images we could observe the total thickness of the photonic crystal, which is around 600 nm.

Transfer matrix method

We present in this paragraph the transfer matrix method employed for the simulation of the transmission of the photonic structure.

In Ref. [1] a detailed description of the transfer matrix method is reported. We have considered The system air/multilayer/glass (in which glass is the sample substrate) and an incidence of the light normal to the stacked layer surface.

We would like to stress that the system is more complex, involving also ITO layers below and on top of the photonic crystal. We neglect this part since it is beyond the scope of this work to deliver an exhaustive understanding of the optical properties of the ITO layers.

n_0 and n_S are, respectively, the refractive indexes of air and glass, while E_m and H_m are the electric and magnetic fields in the glass substrate. In order to determine the electric and magnetic fields in air, E_0 and H_0, we solved the following system:

$$\begin{bmatrix} E_0 \\ H_0 \end{bmatrix} = \prod_{i=1}^{m} M_m \begin{bmatrix} E_m \\ H_m \end{bmatrix} = \begin{bmatrix} m_{11} & m_{12} \\ m_{21} & m_{22} \end{bmatrix} \begin{bmatrix} E_m \\ H_m \end{bmatrix} \quad (1)$$

where

$$M_j = \begin{bmatrix} A_j & B_j \\ C_j & D_j \end{bmatrix}$$

with j=(1,2,…,m). M_j is the characteristic matrix of each layer. The elements of the transmission matrix M_j are

$$A_j = D_j = cos(\phi_j), \quad B_j = -\frac{i}{p_j} sin(\phi_j), \quad C_j = -ip_j sin(\phi_j) \quad (2)$$

The phase variation of the wave passing the j-fold layer is $\phi_j = (2\pi/\lambda) n_j d_j$, where n_j and d_j, contained in the angle ϕ_j, are respectively the effective refractive index and the thickness of the layer j. The coefficient $p_j = \sqrt{\varepsilon_j/\mu_j}$ in transverse electric (TE) wave. Inserting Equation (2) into Equation (1) and using the definition of transmission coefficient,

$$t = \frac{2p_S}{(m_{11}+m_{12}p_0)p_S + (m_{21}+m_{22}p_0)} \quad (3)$$

it is possible to write the light transmission as

$$T = \frac{p_0}{p_s}|t|^2 \quad (4)$$

The refractive index of Ag nanoparticles is described in the manuscript. We have employed the effective medium approximation also for TiO_2 nanoparticles, using the refractive index of TiO_2 as reported in Ref.[2]

Simulation of the transmission

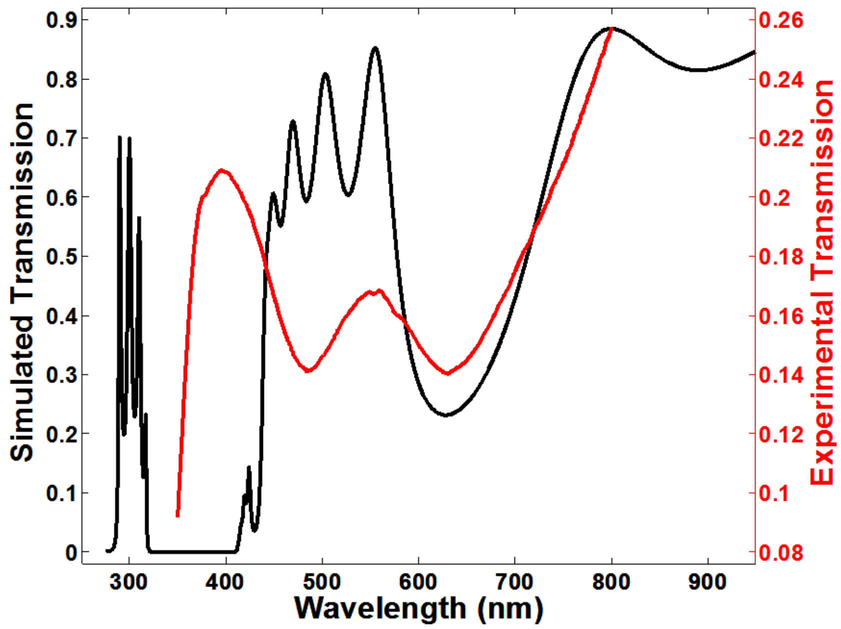

Figure S3: black curve: simulated transmission spectrum of the 5 bilayer Ag/TiO_2 photonic crystal. red curve: experimental transmission spectrum of the 5 bilayer Ag/TiO_2 photonic crystal at 0 V as in Figure 1 of the manuscript.

In Figure S3 the transmission spectrum of the photonic structure is depicted (in black the simulation and in red the experimental data). The parameters for Ag are reported in Reference 27 of the manuscript, while for the photonic structure we have employed the following parameters in the transfer matrix method (please note that in these simulations the EMA has been used to simulate the dielectric properties of both, the Ag and the TiO_2 layers):

- Ag layer thickness: 60 nm
- TiO_2 layer thickness: 105 nm
- Ag filling factor: 50 %
- TiO_2 filling factor: 70 %

The filling factor of the Ag nanoparticle layers is lower since the size of the nanoparticles is larger with respect to TiO_2 (estimation: <50 nm for Ag and <15 nm for TiO_2, respectively). The filling factor of TiO_2 layers is in agreement with previous estimations [2,3].

The Ag nanoparticle/TiO_2 nanoparticle bilayer thickness in the simulation is thus 165 nm, very close to the profilometry measurements of the single layers - deposited directly on the substrate - of 60 nm for the Ag layer and 120 nm for the TiO_2.The SEM cross section measurements instead relate to a bilayer thickness of about 120 nm (1/5 of the total thickness, which is 600 nm). This discrepancy might result from an intermixing of the nanoparticles at the layer interfaces, not taken into account by the simulation. This is also in agreement with the observations made by AFM.

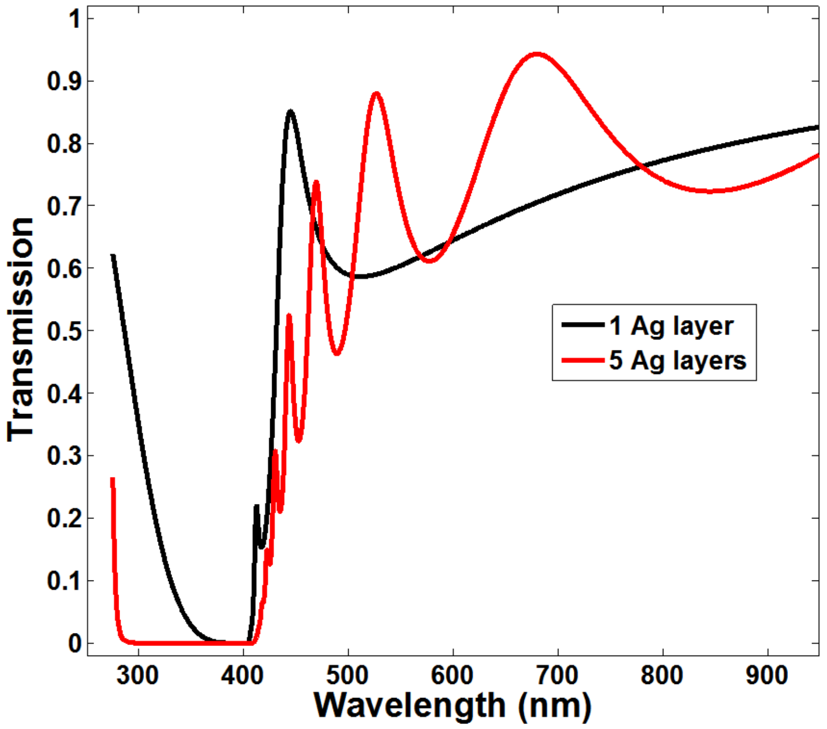

Figure S4: Simulation of the transmission spectrum of a single layer of Ag nanoparticles (black curve) and 5 layers of Ag nanoparticles (red curve).

We show in Figure S4 the calculated transmission spectra of Ag nanoparticle layers considering only the imaginary part of the refractive index of the Ag nanoparticle layer, taking into account only the contribution related to the absorption. 1 Ag layer corresponds to 60 nm, 5 Ag layers to 300 nm. Around 400 nm the transmission gets close to zero, due to the intense absorption of the Ag plasmon resonance, more intense in the 5 layer structure.

For comparison, in Figure S5 we show the transmission spectrum of the entire photonic crystal considering the complete refractive index (real and imaginary components of Ag - black curve) and only the real part of the refractive index (red curve). From these calculations it becomes clear that the intense band around 400 nm is due to the strong absorption of the Ag plasmon resonance, while the second band around 630 nm is due to the photonic bandgap.

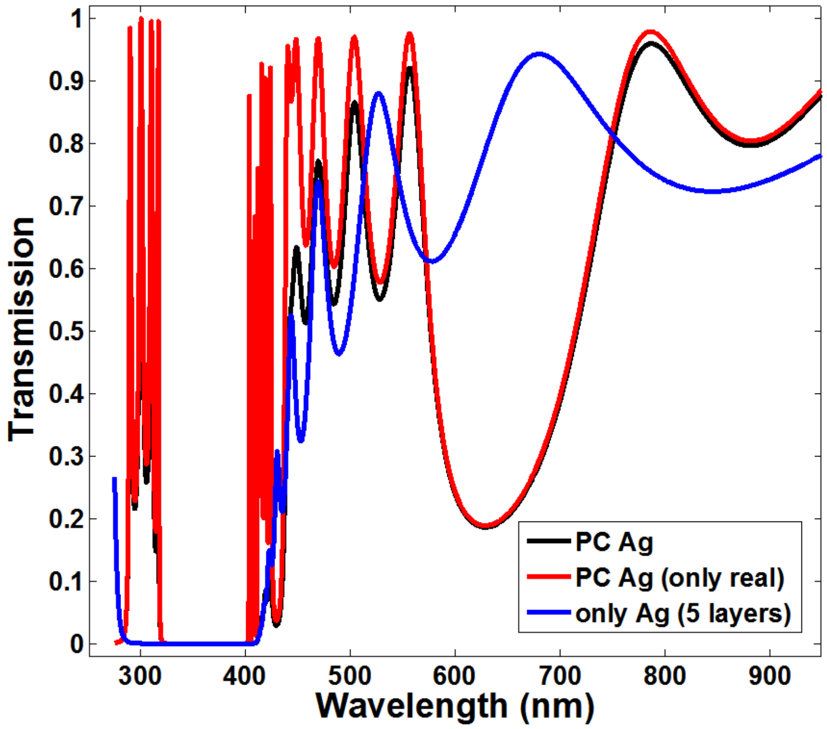

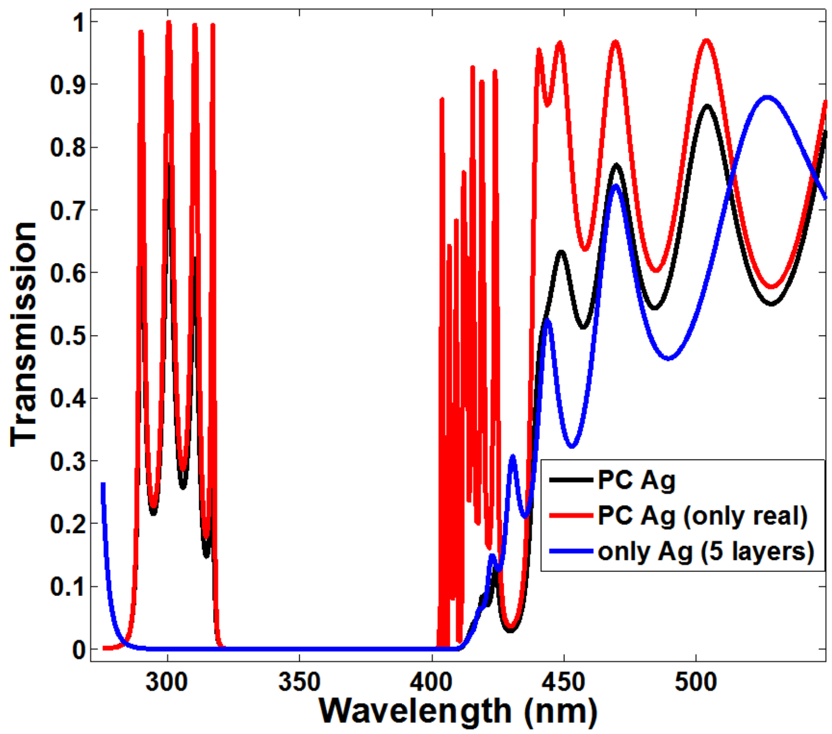

Figure S5: Simulation of the transmission spectrum of: a (Ag nanoparticle/TiO_2 nanoparticle)$_5$ photonic crystal with the refractive index (real and imaginary part) of silver (black curve); a (Ag nanoparticle/TiO_2 nanoparticle)$_5$ photonic crystal with the refractive index (only real part) of silver (red curve); 5 layers of Ag (real and imaginary part of the refractive index, blue curve) as in Figure S4. Top: in the region between 250 and 950 nm. Bottom: in the region between 270 and 550 nm.

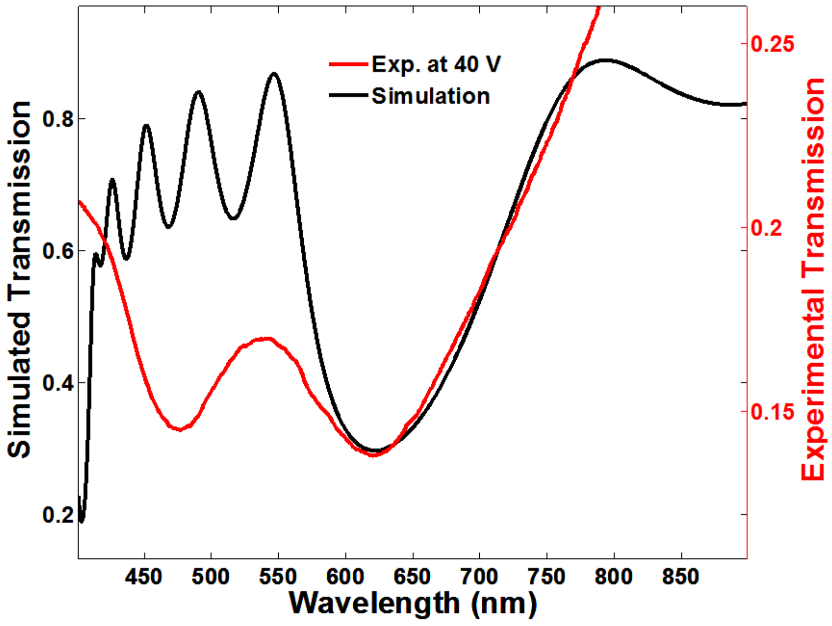

Figure S6: Simulation of the transmission spectrum of the 5 bilayer Ag/TiO_2 photonic crystal at 40 V.

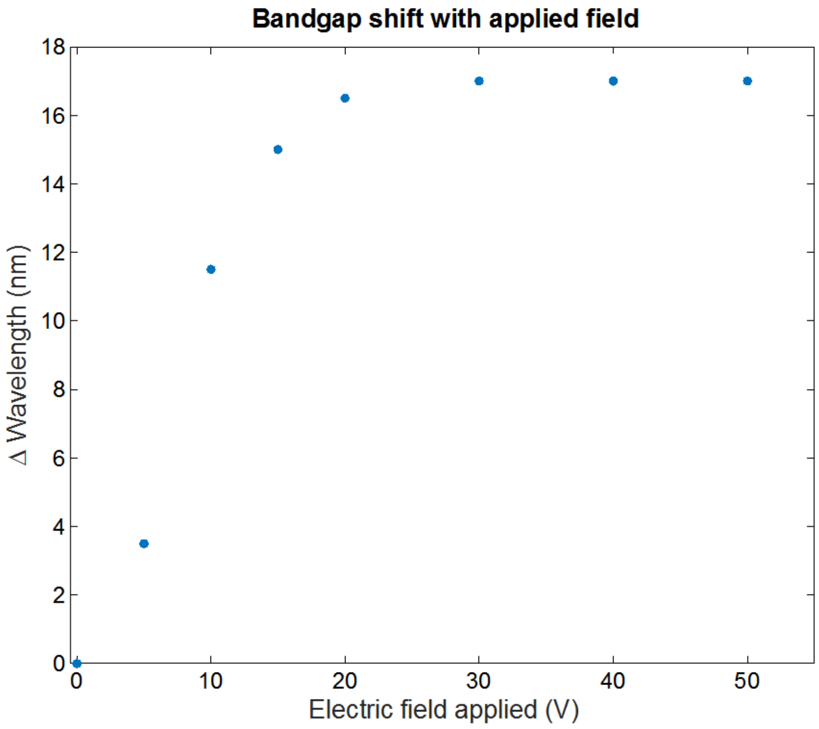

Figure S7: Shift of the blue edge of the photonic band gap as a function of the applied voltage.

References

1. Born, M. & Wolf, E. *Principles of Optics: Electromagnetic Theory of Propagation, Interference and Diffraction of Light*. (CUP Archive, 2000).
2. Scotognella, F. *et al.* Metal oxide one dimensional photonic crystals made by RF sputtering and spin coating. *Ceram. Int.* **41,** 8655–8659 (2015).
3. Puzzo, D. P. *et al.* Color from colorless nanomaterials: Bragg reflectors made of nanoparticles. *J. Mater. Chem.* **19,** 3500–3506 (2009).
